# Impacts of Armed Conflicts on Healthcare and Nutrition Services in Ethiopia: A Narrative Review

**DOI:** 10.1002/puh2.70099

**Published:** 2025-08-09

**Authors:** Kassahun Ayele

**Affiliations:** ^1^ Department of Food and Nutritional Science Wollega University Nekemte Ethiopia

**Keywords:** armed conflicts, healthcare, nutrition services

## Abstract

Armed conflicts in Ethiopia have profoundly impacted healthcare and nutrition services, exacerbating pre‐existing vulnerabilities and introducing new challenges. This narrative review synthesizes peer‐reviewed literature and reports to offer a comprehensive understanding of the effects of war and conflict on healthcare and nutrition services in Ethiopia. The review included peer‐reviewed articles and gray literature published between 2015 and 2025 that focused on healthcare and nutrition services in conflict‐affected regions of Ethiopia. The review underscores the geographical barriers, infrastructure damage, and resource constraints that impede access to healthcare facilities and disrupt essential medical services. Maternal and child health services have been particularly affected, with limited antenatal care, increased maternal mortality, and disrupted vaccination programs. The conflict has also precipitated a severe food crisis, characterized by widespread food insecurity and acute malnutrition among children in conflict zones. Displaced populations face heightened risks of disease and malnutrition due to overcrowded conditions, limited access to water and sanitation, and disrupted livelihoods. The review emphasizes the urgent need for humanitarian assistance, including food, water, shelter, and healthcare, to prevent further deterioration of the situation. Collaborative approaches focusing on peacebuilding and health infrastructure investment are essential for restoring services and improving health outcomes. The review aims to inform policymakers, healthcare providers, and humanitarian organizations about the challenges faced by conflict‐affected communities and the importance of targeted interventions to address the immediate and long‐term needs of these populations.

## 1 Background

Armed conflicts represent a significant global issue, impacting millions and leading to crises such as malnutrition, disease, and increased morbidity [1]. The conflicts disrupt societal structures and destroy agricultural systems, hindering humanitarian aid and jeopardizing the health of those affected [2]. The consequences go beyond immediate physical damage, resulting in enduring socio‐economic effects that obstruct recovery efforts. Political manipulation complicates these issues, affecting water sanitation, increasing gender‐based violence, and causing population displacement [3]. Such manipulation undermines basic services and heightens vulnerability of marginalized groups. The obstruction of humanitarian aid and weaponization of resources intensify civilian suffering. War and armed conflicts continue to burden global health, development, and humanitarian systems, with crises in Ukraine, Syria, Gaza, Sudan, and the Sahel region demonstrating the toll on civilians.

The Horn of Africa, including Sudan and Ethiopia, has experienced escalating strife, deepening the crisis confronting its population [2, 4]. This region's vulnerability stems from political instability resource scarcity, and ethnic tensions, contributing to a protracted humanitarian emergency. These conflicts undermine development efforts and perpetuate cycles of violence and displacement. Access to healthcare and nutrition services is critical, yet often compromised in conflict zones [5]. The disruption leads to increased morbidity and mortality rates, particularly among women and children. Targeting of healthcare facilities and personnel denies communities access to life‐saving care. Public health systems experience extensive damage, facing shortages of medications, supplies, and transportation challenges [6]. The breakdown of health infrastructure undermines response to disease outbreaks and essential care.

Ethiopia has faced political instability and complex challenges over 5 years [7]. These include internal conflicts, ethnic tensions, and socio‐economic disparities, creating a volatile environment. The 2020 war between Tigray forces and the Ethiopian National Defense Forces (ENDF) escalated into a regional conflict, causing a humanitarian crisis. The conflicts have resulted in a humanitarian crisis, necessitating assistance, psychological support, and resources to rebuild livelihoods [1]. The displacement of communities, destruction of infrastructure, and loss of livelihoods have created a dire humanitarian situation, requiring a coordinated response to address immediate needs of affected populations. The provision of psychological support is crucial to address trauma and mental health challenges experienced by individuals affected by violence and displacement. Prior to the conflicts, the Ethiopian government had implemented nutritional policies and programs; however, these initiatives have deteriorated amidst the violence [8]. The disruption of services and diversion of resources towards conflict‐related activities have undermined these programs, leading to increased malnutrition and food insecurity. The restoration of these nutritional policies and programs is essential to mitigate the long‐term consequences of conflict on public health and nutrition.

This review aimed to synthesize literature and reports concerning the impact of war and conflict in Ethiopia, particularly their effects on nutrition and public healthcare services. By bringing together diverse sources, the review aims to provide a comprehensive understanding of the challenges faced by communities affected by conflict. The focus on nutrition and public healthcare services recognizes the interconnectedness of these critical aspects of human well‐being. The review informs policymakers, healthcare providers, and humanitarian organizations about challenges faced by communities in conflict, emphasizing the need for targeted interventions to address healthcare and nutritional needs. By providing evidence‐based insights, the review aims to contribute to effective policies and programs that address the needs of conflict‐affected populations. The dissemination of findings to stakeholders is essential to promote collaboration and action.

## 2 Methods

### 2.1 Literature Search Strategy

This narrative review was conducted to synthesize existing literature on the impact of armed conflicts on healthcare and nutrition services in Ethiopia. A comprehensive literature search was conducted using electronic databases, including PubMed, Scopus, Web of Science, and Google Scholar, covering publications from January 2015 to March 2025 to capture recent developments. The search strategy employed key terms and Boolean combinations such as “armed conflict” AND “Ethiopia” AND (“health services” OR “nutrition services” OR “healthcare system” OR “nutrition programs”).

### 2.2 Inclusion and Exclusion Criteria

The review prioritized peer‐reviewed articles, systematic reviews, meta‐analyses, and commentaries, as well as gray literature such as reports from governmental bodies, UN agencies, reputable NGOs, and civil society organizations. Studies were included if they provided empirical data and relevant contextual information on health and nutrition services in conflict‐affected settings in Ethiopia. Only publications in English were considered. Duplicate studies, non‐relevant topics, and articles without accessible full texts were excluded. This structured approach was intended to minimize bias and ensure a comprehensive and balanced synthesis of the available evidence.

## 3 Impacts of Conflicts on Healthcare and Nutrition Services

### 3.1 Impact on Healthcare Access and Infrastructure

In Ethiopia, accessing primary healthcare facilities presents significant challenges due to their remote locations from urban centers [9]. The geographical spread in rural areas complicates access to essential medical care, particularly in underserved regions. Limited transportation infrastructure and challenging terrain further hinder timely access to healthcare facilities. These geographical barriers complicate the transportation of medical supplies and equipment, affecting the continuity of public health services [10]. Delivering essential supplies demands substantial resources, which are scarce in conflict‐affected areas. Insecurity and road closures disrupt transportation routes, leading to supply shortages.

Healthcare facilities under the control of armed opposition groups disrupt the distribution of health resources and the provision of medical care to civilians [6, 11]. This occupation restricts patient access and endangers healthcare workers. The diversion of medical supplies for military purposes depletes resources meant for civilian healthcare. Armed conflicts in Ethiopia have damaged healthcare infrastructure, resulting in adverse health outcomes [12, 13]. The destruction of healthcare facilities reduces medical service capacity. Disruptions in the supply chain, displacement of healthcare workers, and the breakdown of public health programs increase mortality rates. In conflict‐affected regions, maternal health services show that only 36.5% of women receive adequate antenatal care [14, 15]. This leads to increased maternal mortality and poor reproductive health outcomes, exacerbated by sexual violence and medical supply shortages.

The war in northern Ethiopia has devastated Tigray's healthcare system, with only 27.5% of hospitals, 17.5% of health centers, and 11% of ambulances remaining functional after 6 months [16]. This collapse has rendered maternal and child health services virtually unavailable. In Amhara, more than 51% of health facilities have been damaged or looted, limiting access to medical care [17]. Essential services for chronic disease management have been interrupted for over 10,000 patients, while maternal and child health services are disrupted for more than 70,000 mothers [18]. In Oromia, over 1072 healthcare facilities have been affected [12]. This breakdown, combined with the evacuation of healthcare personnel and shortages of medical supplies, has critically hindered healthcare provision. Healthcare facilities have suffered extensive damage in conflict‐affected areas, undermining the system's capacity to meet the population's needs [19]. The destruction reduces the availability of medical services and disrupts the supply of essential medicines. The damage to health facilities in Amhara has particularly impacted rural areas where alternative care sources are limited [17]. This destruction also undermines healthcare worker morale, affecting their willingness to work in unsafe conditions.

In Oromia, the extensive damage to healthcare facilities highlights the urgent need for reconstruction efforts [20]. The service disruption significantly impacts population health, especially in rural areas with limited alternative care options. Child health services have suffered, with 39% of infants missing essential vaccinations during the conflict period, raising concerns about disease [21]. The disruption of vaccination programs increases the risk of outbreaks like measles, polio, and diphtheria, undermining children's long‐term health and making them vulnerable to illness. Limited health services correlate with infectious diseases, evidenced by 7.3 million malaria cases and 1157 deaths, highlighting children's vulnerability in conflict settings [22]. Limited healthcare access and poor sanitation increase infection risks, particularly among displaced populations in overcrowded conditions. The collapse of maternal and child health services in Tigray has made antenatal care, deliveries, and vaccinations unavailable [15, 16]. This disruption has increased maternal mortality, infant mortality, and malnutrition rates, making service restoration essential for improving health outcomes.

### 3.2 Impact on Food and Nutrition Security

Ethiopia, Africa's second most populous nation and third largest refugee host, is grappling with a severe food crisis exacerbated by ongoing conflicts [23]. This crisis has led to widespread hunger and food insecurity, particularly impacting populations in conflict zones. Conflicts disrupt food production, transportation, and distribution, heightening the risks of food insecurity and starvation [24]. Agricultural activities have diminished, and damaged infrastructure hinders the transportation of food to those in need. Nutritional deficiencies contribute to undernutrition and increased vulnerability to disease, perpetuating cycles of poverty and ill‐health. Undernutrition weakens immunity and hampers development, especially in children, with long‐lasting health consequences. Children in conflict areas suffer severe health impacts due to limited access to essential services [11]. Conflict‐induced food shortages and price hikes, coupled with displacement and loss of livelihoods, elevate the risks of child malnutrition. Studies indicate that 41.23% of children are acutely malnourished, and two‐thirds of children are acutely malnourished in conflict zones [25, 26]. Acute malnutrition heightens the risk of illness and necessitates urgent therapeutic intervention. The armed conflict in northern Ethiopia since November 2020 has significantly reduced the efficacy of food security programs, undermining efforts to combat hunger and malnutrition [24]. Program disruptions, resource diversions, and beneficiary displacement have contributed to this decline, creating barriers to monitoring and evaluation. The Productive Safety Net Program (PSNP), which provides cash and food transfers to vulnerable households in exchange for participation in public works, has experienced reduced funding amid the turmoil, exacerbating food insecurity [24]. This reduction has impacted the program's ability to reach those in need, particularly in conflict‐affected areas. Violence continues to affect food security across Ethiopia, underscoring the need for sustainable solutions and humanitarian efforts [8]. Conflicts disrupt agriculture, displace communities, and undermine livelihoods, impeding food access. Solutions must address the root causes of conflict while meeting immediate food security needs.

### 3.3 Conflict‐Induced Displacement and Consequences

The conflict has displaced over 1.55 million people from Amhara, leading to a humanitarian crisis and overwhelming the support capacity of host communities [18]. This displacement disrupts access to essential services, heightening population vulnerability and placing additional burdens on host communities. Displaced populations endure overcrowded conditions lacking in hygiene, sanitation, and nutrition, which elevates the risks of disease and malnutrition [27]. These conditions facilitate the spread of respiratory infections, diarrheal diseases, and skin infections. Limited access to water, sanitation, and hygiene exacerbates disease risks, particularly among children and pregnant women. Children, women, and the elderly are especially vulnerable to the effects of displacement. Children face heightened risks due to their immature immune systems, whereas women encounter challenges related to pregnancy and reproductive health. Elderly individuals’ pre‐existing conditions worsen without access to healthcare.

Ethiopia is grappling with a humanitarian crisis, with 21.4 million people, including 12 million children, in need of assistance [26]. This crisis, driven by conflict, drought, and displacement, necessitates a coordinated response from both government and international organizations. The armed conflict in Amhara has precipitated a public health emergency, causing direct impacts such as injuries and displacement, as well as indirect effects like healthcare disruption and food insecurity [26]. The crisis demands immediate and long‐term health interventions. The displacement has forced 1.55 million individuals into overcrowded internally displaced persons (IDP) sites lacking basic necessities [28]. This situation disrupts access to essential services and heightens disease risks, particularly affecting children and pregnant women. The disruption of access to essential services, such as healthcare, food, and water/sanitation, severely impacts poorer households that lack the resources to cope with displacement. These households rely more on public services and are vulnerable to service disruptions, particularly affecting children and elderly members.

Families with undernourished children and rural communities face more severe impacts from conflict and displacement. These groups experience greater food insecurity and direct exposure to conflict areas. Wealthier and urban households experience less severe impacts due to their access to private healthcare, alternative food supplies, and improved sanitation [29, 30]. Their resources and proximity to diverse services provide protection against disruptions. IDPs face higher disease rates due to limited access to healthcare, including preventive care and treatment [27]. This is a result of destroyed facilities, healthcare worker shortages, and disrupted supply chains. Children and pregnant women in IDP sites face elevated risks of malnutrition, infectious diseases, and pregnancy‐related complications. Their vulnerability necessitates targeted interventions. Ethiopia's conflict zones face critical nutritional challenges, with IDPs experiencing severe food insecurity and malnutrition [31]. Conflict has disrupted food systems and access to clean water, particularly affecting children.

### 3.4 The Role of Humanitarian Aid and Intervention Strategies

Millions of Ethiopians living in conflict areas are in desperate need of humanitarian aid, including food, water, shelter, and healthcare [32]. This crisis demands a coordinated international effort to deliver life‐saving support to those affected. In Amhara, around 21.3 million individuals urgently need food assistance to avert starvation [28]. The food crisis has been exacerbated by agricultural disruptions, displacement, and the destruction of infrastructure. Additionally, refugees from Sudan seeking aid are putting a strain on limited resources, necessitating a coordinated response to address the needs of both refugees and host communities. In Oromia, challenges, such as insecurity, logistical issues, and bureaucratic hurdles, hinder the effective distribution of aid [12]. Conflicts have disrupted transportation routes and posed security threats to humanitarian workers, whereas limited storage capacity and inadequate infrastructure further impede aid delivery. Violence has damaged public health infrastructure, leading to increased malnutrition among IDPs and pregnant women. The destruction of healthcare facilities, shortages of workers, and disrupted supply chains have undermined essential medical services. Limited access to water and sanitation has heightened infection risks among displaced populations.

## 4 Limitations of Current Review and Future Directions

Focusing solely on health and nutrition services might neglect other crucial services affected by conflict, such as education and sanitation, potentially limiting the understanding of the full impact of armed conflict on communities. Although health and nutrition are vital to human well‐being, conflict also disrupts other essential services, including education, sanitation, and livelihoods. A more comprehensive analysis of conflict's impact would consider the interconnections among these sectors and their collective effect on communities. Further research is necessary to gain a holistic understanding of the multifaceted challenges these communities face, capturing the diverse experiences and perspectives of those affected by conflict and ensuring that interventions are culturally sensitive and contextually appropriate.

## 5 Conclusion

The armed conflict in Ethiopia has severely affected health and nutrition services, compromising the well‐being and increasing the vulnerabilities of the population. Health facilities are experiencing a decline in operational capacity, with healthcare personnel facing risks that restrict the provision of essential services. This disruption has led to deteriorating health outcomes, especially among vulnerable groups such as women, children, and displaced persons. It is crucial for organizations to prioritize the continuity of health services in conflict zones, ensuring that those most in need have access. Implementing mobile health clinics, deploying community health workers, and establishing secure supply chains are necessary to reach isolated communities. Collaborative efforts that focus on peacebuilding and investment in health infrastructure are vital for restoring services and improving outcomes. Stakeholders must commit to peace and address the root causes of the conflict. Humanitarian agencies need to provide essential assistance to ensure that affected populations have access to basic necessities. Investigations should assess the impact of the conflict across health, education, and economic sectors to inform interventions and guide peacebuilding initiatives.

## Author Contributions

Kassahun Ayele conceptualized the study idea, performed the literature search, selected the article of interest, performed data extraction, read, revised, and approved the manuscript.

## Ethics Statement

The author has nothing to report.

## Consent

The author has nothing to report.

## Conflicts of Interest

The author declares no conflicts of interest.

## Data Availability

All data generated during this review are included in the submitted manuscript.
